# USING COMMUNITY-BASED PARTNERSHIPS TO SUPPORT ALZHEIMER’S DISEASE AND RELATED DEMENTIAS PROGRAMS

**DOI:** 10.1093/geroni/igae098.1304

**Published:** 2024-12-31

**Authors:** Samantha Cotton, Monica Long

**Affiliations:** University of Louisville, Louisville, Kentucky, United States; University of Chicago Medicine/SHARE Network, Glenwood, Illinois, United States

## Abstract

Launched in 2015, the University of Chicago’s SHARE Network and the University of Louisville’s Geriatric Workforce Enhancement Program (GWEP) showcase transformative approaches to aging and dementia care through dynamic community and state partnerships. SHARE Network addresses the needs of Chicago’s South Side, aiming to mitigate health disparities through collaborations that have led to the development of resources like memory cafes and caregiver support groups. This initiative enriches healthy aging and dementia education among healthcare professionals and community organizations.Similarly, the University of Louisville’s GWEP focuses on ensuring accessible services via collaborations with Area Agencies on Aging, senior centers, and other service organizations, alongside strategic partnerships with Kentucky’s health departments. These efforts underscore the development of career pathways for Community Health Workers and Certified Nursing Assistants, supporting workforce development and enhancing dementia care. In this presentation, we will delve into the critical need for community partnerships in dementia care, highlighting how the SHARE Network and the University of Louisville’s GWEP have successfully harnessed these relationships to improve care for those affected by dementia. We will also explore the challenges and barriers encountered in implementing these programs and discuss strategies to overcome them. By sharing insights on engaging diverse stakeholders, navigating systemic obstacles, and leveraging resources for maximum impact, the presentation aims to provide a comprehensive overview of how to strengthen dementia care through effective community collaboration, ultimately leading to improved outcomes for individuals with dementia and their caregivers.

